# Quality Improvement Project on Standardising GP Discharge Summaries in Liaison Psychiatry Services for Older People in Nottinghamshire Healthcare NHS Trust

**DOI:** 10.1192/bjo.2024.443

**Published:** 2024-08-01

**Authors:** Sue Fen Tan, Sarah Wilson, Robin Lander

**Affiliations:** 1 Nottinghamshire Healthcare NHS Trust, Nottingham, United Kingdom; 2 Institute of Mental Health, Nottingham, United Kingdom

## Abstract

**Aims:**

Discharge letters to general practitioners (GPs) are pertinent in summarising patients' care in secondary healthcare settings and communicating follow-up management plans for continuity of care. 26 GPs from 13 GP surgeries in the West Midlands thought that discharge letters lacked important information and standardisation. We developed a quality improvement (QI) project to standardise GP discharge summaries within the liaison psychiatry services for older people in Nottinghamshire Healthcare NHS Trust. We aimed to ensure that 100% of GP discharge letters are written in a standardised format and meet the mandatory subheadings within six months.

**Methods:**

A comprehensive literature search was performed, and we invited six GPs across Nottinghamshire to comment on the quality of anonymised discharge summaries written by our colleagues. After discussing the findings with our stakeholders, we developed a new discharge summary template with the subheadings of ‘Reason for Liaison Psychiatry Involvement’, ‘Summary’, ‘Diagnosis (if applicable)’, ‘Risk Formulation’, and ‘Treatment or Plan of Action’.

We held a team meeting and distributed a guidance document with scoring criteria for each subheading for our clinical colleagues to practise for two weeks. Subsequently, 75 discharge summaries were randomly selected and independently scored across seven weeks by an internal team member and an external QI data analyst to improve inter-rater reliability. 98 discharge summaries written six weeks before the new letter template was introduced were retrospectively scored for baseline measurement.

**Results:**

At baseline, the discharge summary scores ranged between 6 and 20 (out of a maximum of 20), depending on the individual completing them. The mean score was 12.3.

The implementation of the new discharge summary template improved the mean score to 19.0, irrespective of the author. The mean score was consistent across seven weeks.

Most of our colleagues did not face significant challenges in learning a new style of writing and for some, a standardised template reduced administrative time. The same GPs reviewed the new set of anonymised discharge summaries and were satisfied with the new summary format.

**Conclusion:**

Formulating a standardised discharge summary template which adhered to professional guidelines was pivotal in improving the quality of GP discharge summaries. GP involvement throughout the project convinced stakeholders and colleagues to commit to a new writing template and tremendously helped achieve our project aim.

